# Prominin 1/CD133 Endothelium Sustains Growth of Proneural Glioma

**DOI:** 10.1371/journal.pone.0062150

**Published:** 2013-04-25

**Authors:** Bi-Sen Ding, Daylon James, Rajiv Iyer, Ilaria Falciatori, Dolores Hambardzumyan, Su Wang, Jason M. Butler, Sina Y. Rabbany, Adília Hormigo

**Affiliations:** 1 Ansary Stem Cell Institute, and Department of Genetic Medicine, Weill Cornell Medical College, New York, New York, United States of America; 2 Brain Tumor Center, Memorial Sloan-Kettering Cancer Center, New York, New York, United States of America; 3 Department of Neurosurgery and Brain Tumor Center, Memorial Sloan-Kettering Cancer Center, New York, New York, United States of America; 4 Department of Neurology, Center for Translational Neuromedicine, Oncology and Neurosurgery, University of Rochester Medical Center, Rochester, New York, United States of America; 5 Bioengineering Program, Hofstra University, Hempstead, New York, United States of America; University of Florida, United States of America

## Abstract

In glioblastoma high expression of the CD133 gene, also called Prominin1, is associated with poor prognosis. The PDGF-driven proneural group represents a subset of glioblastoma in which CD133 is not overexpressed. Interestingly, this particular subset shows a relatively good prognosis. As with many other tumors, gliobastoma is believed to arise and be maintained by a restricted population of stem-like cancer cells that express the CD133 transmembrane protein. The significance of CD133^+^ cells for gliomagenesis is controversial because of conflicting supporting evidence. Contributing to this inconsistency is the fact that the isolation of CD133^+^ cells has largely relied on the use of antibodies against ill-defined glycosylated epitopes of CD133. To overcome this problem, we used a knock-in *lacZ* reporter mouse, *Prom1^lacZ/+^*, to track Prom1^+^ cells in the brain. We found that Prom1 (prominin1, murine CD133 homologue) is expressed by cells that express markers characteristic of the neuronal, glial or vascular lineages. In proneural tumors derived from injection of RCAS-PDGF into the brains of tv-a;*Ink4a-Arf^−/−^ Prom1^lacZ/+^* mice, Prom1^+^ cells expressed markers for astrocytes or endothelial cells. Mice co-transplanted with proneural tumor sphere cells and Prom1^+^ endothelium had a significantly increased tumor burden and more vascular proliferation (angiogenesis) than those co-transplanted with Prom1^−^ endothelium. We also identified specific genes in Prom1^+^ endothelium that code for endothelial signaling modulators that were not overexpressed in Prom1^−^ endothelium. These factors may support proneural tumor progression and could be potential targets for anti-angiogenic therapy.

## Introduction

Glioblastoma (GBM) is the name given to the most common and aggressive primary brain tumor of adults. Although histologically identical, different subtypes of glioblastoma can be identified by immunohistochemical and genetic analysis and correlate with different prognoses [1], [2], [3]. Molecular classification identifies 3 or 4 GBM subclasses [1], [2], [3]. One subtype, the proneural GBM occurs in patients who are usually younger, have longer survival and have tumors enriched in PDGFA receptor [2] andOlig2 [3]. CD133 is a marker of neural stem cells and of a unique population of rare cells, believed to be “cancer stem cells”. CD133 is found in many malignant tumors, including glioblastoma [4], [5] and is highly expressed in poor prognosis subtypes along with markers of proliferation and angiogenesis [1], [2], [3]. However, CD133 is not believed to be a signature of the proneural subclass [1]. Microvascular proliferation is a histologic characteristic of all subtypes of GBMs and CD133 is expressed by the vascular structures in these tumors [6]. In a glioma mouse model induced by human PDGFb, CD133 expressing cells were among recruited cells and were not derived from the progeny of glioma cell-of-origin [7].

CD133/Prom1/AC133 is a cholesterol binding pentaspan membrane glycoprotein that localizes to microvilli or cilia in the apical domain of epithelial and non-epithelial cells [8], [9]. It is conserved among different species [10] and it is expressed as tissue-specific splice variants both in human [11] and in mouse [12]. The biological function of the protein remains largely unknown, although lack of Prom1 has been linked to degeneration of photoreceptors and vision loss [13]. In normal brain, CD133^+^ stem cells reside in the subventricular zone (SVZ) and in the hippocampal subgranular zone (SGZ) neural and vascular niches [14], [15] and are thought to be maintained by growth factors, such as pigment epithelium-derived factor (PEDF) [15], [16] and brain-derived neurotrophic factor (BDNF) [16]. CD133 positive cells identified in many malignant tumors including glioblastoma are believed to be cancer stem cells, a subset of malignant cells that are resistant to most therapeutic endeavors. Survival of these cells after treatment is believed to lead to early recurrence of the glioblastoma. The identification of the cells has been based on antibody recognition of posttranslational modifications of CD133 protein, however the expression of the glycosylated epitopes can be variable and even absent [17] and therefore this technique can lead to discrepancies in determining organ and cell-lineage specific expression pattern of Prom1/CD133 [18], [19], [20]. The lack of an operational marker and faithful or authentic genetic reporter greatly limits the identification of the mechanistic role of CD133 cells as brain stem-like cells and endothelial progenitors. To study the contribution of CD133 to proneural GBM subgroup formation and elucidate the intertwined relation between CD133^+^ neural stem cells and vasculature *in vivo,* we used a mouse model in which the reporter gene *LacZ* was introduced in the *Prom1* locus under control of *Prom1* promoter [12], thus avoiding the limitations created by deficient recognition of a functional group on CD133 protein. We found that Prom1 is expressed by cells that have morphological phenotypes and express markers for neurons, astrocytes, neural progenitor cells, ependyma or endothelial cells in the normal adult brain. We also found that in proneural GBM-like tumors, Prom1 is expressed by endothelium. In these tumors, Prom1endothelium supports microvascular proliferation and accelerates tumor growth by producing biologically active factors that may promote progression. These factors should be considered potential targets in the development of anti-angiogenic therapies.

## Results

### Prom1 is Widely Expressed in the Adult Brain

**Figure 1:**
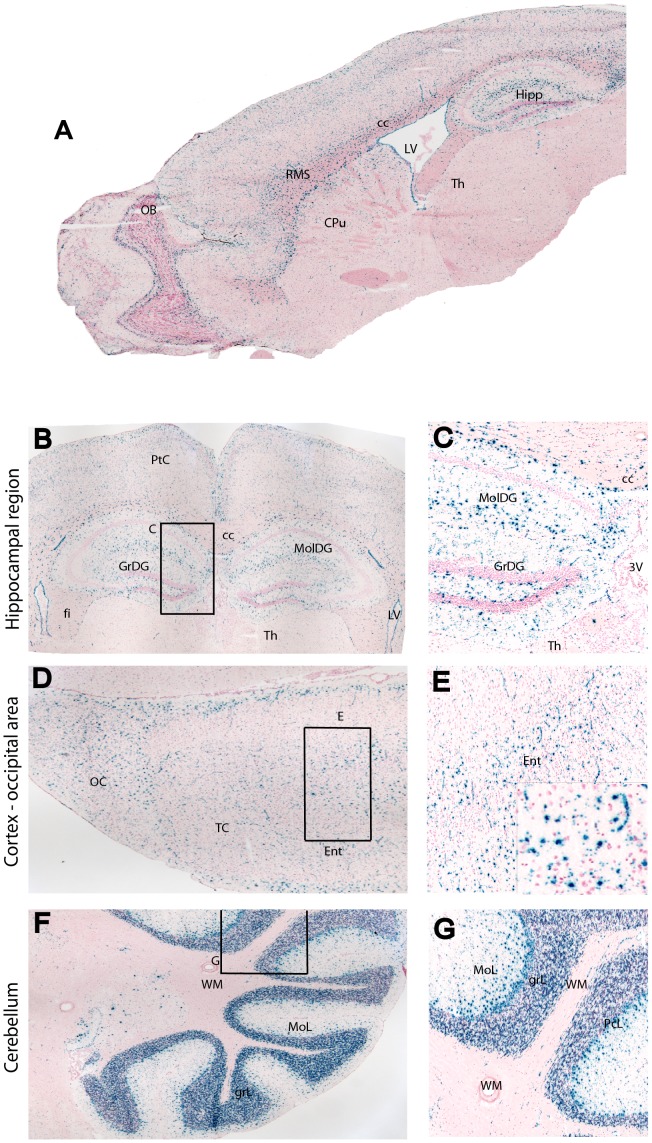
Prom1^+^ cells are present throughout the brain of an eight-week old *Prom1^lacZ/+^* mouse model. (A) X-gal staining of Prom1 cells in sagittal section of the brain. (B–F, left panel) Low-power and respective magnified images (C-G, right panel) of anteroposterior coronal sections showing the distribution of ß-galactosidase activity in the hippocampus (B, C), occipital cortex (D, E) and cerebellum (F, G). Abbreviations: CPu, caudate/putamen; Ent, enthorinal cortex; fi, fimbria; GrDG, granular layer dentate gyrus; GrL, granular layer cerebellum; hipp, hippocampus; LV, lateral ventricle; MolDG, molecular layer dentate gyrus; MoL, molecular layer cerebellum; OB, olfactory bulb; OC, occipital cortex; PcL, Purkinje cell layer; RMS, rostral migratory stream; Tc, temporal cortex; Th, thalamus; WM, white matter; 3V, third ventricle.

To determine the distribution of Prom1 cells in the mouse brain, we detected ß-galactosidase activity by using X-gal staining in the *Prom1^lacZ/+^* mouse brain. Compared to other antibody-based isolation and detection, this mouse line carries ß-galactosidase driven by the endogenous *Prom1* promoter, thereby providing faithful tracking of Prom1^+^ cell lineage. The Prom1^+^ cells were found throughout the entire brain (Fig. 1 and Figure S1). They were common along the rostro-migratory pathway (Fig. 1 A and Figure S1A), subventricular zone (SVZ), hippocampal dentate gyrus (Fig. 1 B and C), Purkinje cell layer and adjacent granular and molecular layers and less common in the white matter and nuclei of the cerebellum (Fig. 1 F and G). Prom1^+^ cells also lined the ependymal epithelium of the lateral (Figure S1 C and D), third and fourth ventricles and aqueduct.

### Prom1 Protein is Expressed by Cells with Neuronal, Astrocytic and Endothelial phenotypes

**Figure 2:**
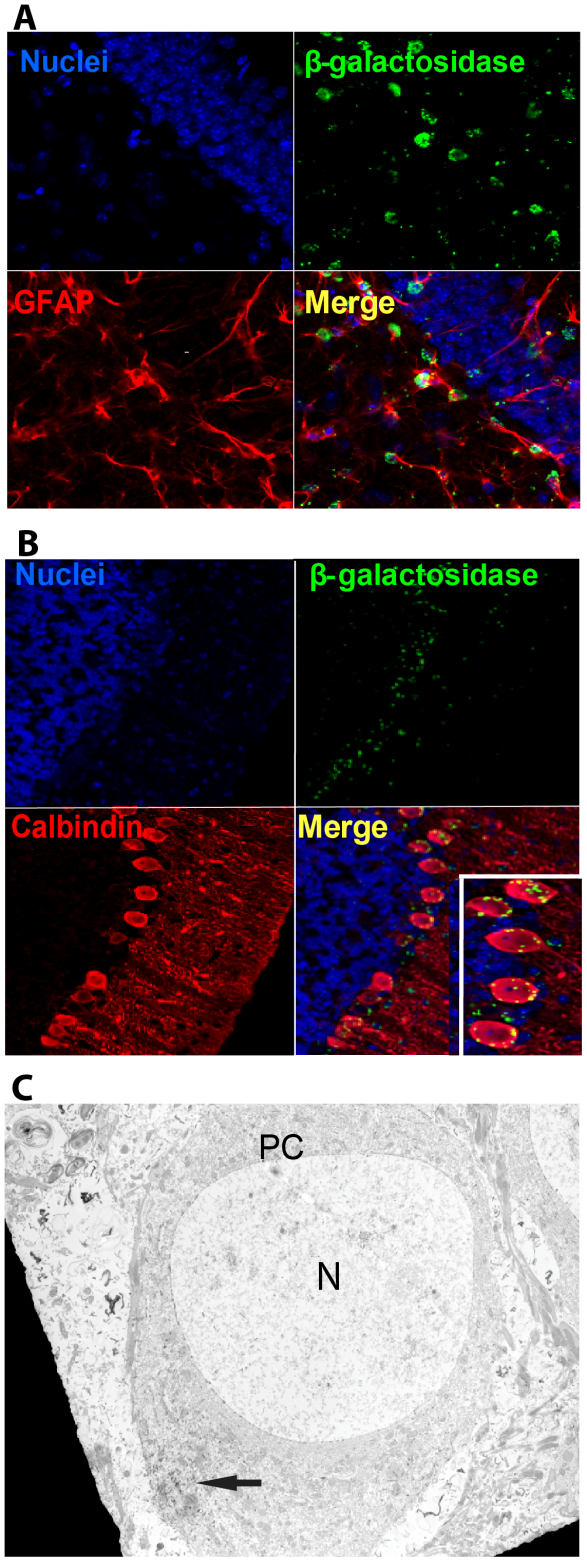
Prom1^+^ neural cells are distributed in the hippocampus and cerebellum of *Prom1^lacZ/+^* in an eight-week old mouse. (A) Z-stack confocal image showing coimmunostaining for ß-galactosidase (green) and GFAP (red) astrocytic cells in the hippocampus and (B) calbindin (red) Purkinje cells in the cerebellum. (C) Electron micrograph shows electron-dense X-gal crystals (arrow) localized in the cytoplasm of Purkinje cells. Abbreviations: N, nucleus; PC, Purkinje cell.

To assess the subset of cells in the brain that express prominin-1, confocal microscopy was used to analyze co-immunostained sections of the *Prom1^lacZ/+^* mouse brain with neural cells specific markers and anti-ß-galactosidase monoclonal antibodies. A subset of ß-galactosidase expressing cells in the olfactory bulb were calretinin^+^ (Figure S2A), a characteristic of post-mitotic interneurons. We detected a large number of prominin-1^+^ cells within the rostral migratory stream (RMS) that co-immunostained for ß-galactosidase and doublecortin (DCX) (Figure S2B), a marker for neurogenesis and migrating neuroblasts. ß-galactosidase expressing cells were also found in the SVZ (Figure S2C) and hippocampus and showed co-staining for glial fibrillary acidic protein (GFAP) (Fig. 2 A), a marker for astrocytes and also known to label neural stem cells. ß-galactosidase expressing cells found within the Purkinje cell layer in the cerebellum exhibited co-immunostaining for calbindin, a neuronal marker for those cells in the cerebellum (Fig. 2 B). We confirmed by electron micrographs that electron-dense X-gal crystals localized to the cytoplasm of Purkinje cells in the cerebellum (Fig. 2 C). In a detailed inspection, we found rare co-localization of ß-galactosidase with Olig2 in the SVZ and hippocampus. There was no co-localization of ß-galactosidase with CNPase, which detects myelin. To characterize ß-galactosidase expressing cells residing in the VZ and SVZ, we performed confocal analysis and found co-immunostaining for ß-galactosidase and CD24, a surface protein expressed by ependymal cells (Figure S2D). The expression of ß-galactosidase in ependyma was further validated by obtaining electron micrographs that revealed electron-dense X-gal crystals in the cytoplasm of the ependymal cells (Figure S2H and I). In addition to the presence of these electrodense granules in the ependyma, they were also found in SVZ astrocytes.

To elucidate whether primary precursor cells express ß-galactosidase, we analyzed both the rapidly and slowly dividing cells in SVZ and olfactory bulb. Mice received BrdU (1 g/L) in drinking water for two weeks followed by three weeks without BrdU [21]. In mice sacrificed immediately after treatment, we found cells that co-immunostained for both anti-ß-galactosidase and anti-BrdU antibodies (Figure S2E), whereas those sacrificed three weeks after the cessation of BrdU administration, there was no co-immunostaining (Figure S2F). These findings suggest that cells that co-express ß-galactosidase and BrdU in the SVZ are rapidly dividing cells, such as transient amplifying progenitor cells that generate olfactory bulb neuronal cells. Some of these progenitor cells might still have remaining morphological characteristics of their cell of origin and contain residual GFP. The absence of co-immunostaining for ß-galactosidase and BrdU in ependymal cells at two weeks (Figure 2 SG), excluded prominin-1^+^/CD24^+^ as transient amplifying cells.

**Figure 3:**
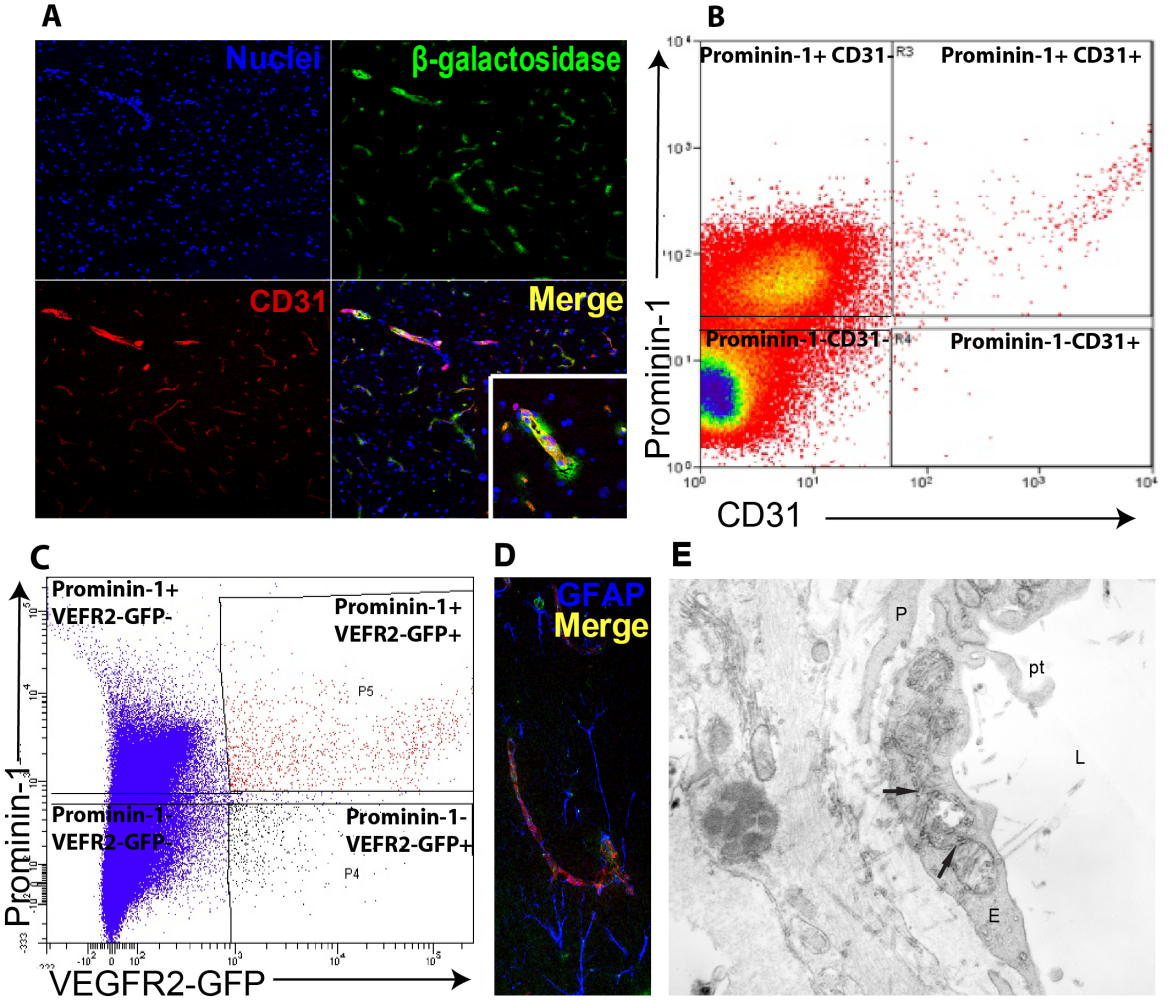
Endothelial cells are among the Prom1^+^ cell population in the brain. (A) Z-stack confocal image showing co-immunostaining for ß-galactosidase (green) and CD31 endothelial cells (red). (B) Flow cytometry confirms the presence of CD31 endothelial cells among Prom1^+^ cells in the brain of a six-week wild-type mouse. (C) Likewise, Prom1^+^ cells are identified among VEGFR2 expressing cells in a reporter mouse in which VEGFR2 promoter drives GFP. CD31 endothelial cells (red) appeared either isolated or (D) wrapped around astrocytes (blue) in the VZ and SVZ areas. (E) Electron micrograph section of *Prom1^lacZ/+^* mouse brain corresponding to areas previously identified by X-gal staining showing rod-shaped electron-dense structures in the endothelial cell cytoplasm of a representative capillary vessel. Arrows, X-gal crystals; E, endothelial cell; L, lumen; P, pericyte; Pt, protrusion.

To delineate the relation of those ß-galactosidase expressing cells within the brain vascular niche, we co-immunostained sections with anti-ß-galactosidase antibody and anti-CD31 antibody, a marker of endothelial cells. We found that a subset of CD31 cells express Prom1 (Fig. 3 A). Triple staining using anti-ß-galactosidase antibody, anti-CD31 antibody and anti-GFAP showed that some Prom1^+^ CD31^+^ vessels were wrapped along processes of astrocytic cells (Fig. 3 D). Next we used flow cytometric analysis to verify the endothelial identity of a subpopulation of Prom1^+^. There were 0.5% of CD31-positive brain endothelial cells that co-expressed Prom1 (Fig. 3 B). To further confirm this observation we decided to use VEGFR2, another endothelial marker. Using fluorescence activated cell sorting (FACS) on cells isolated from the brain of transgenic mice expressing green fluorescent protein (GFP) under control of the VEGFR2 promoter, we found that the subpopulation of VEGFR2-GFP endothelial cells co-expressing Prom1 represents 0.3±0.06% (N = 5) (Fig. 3 C). We also confirmed, using electron microscopy, that a subset of capillary endothelial cells in the brain expresses Prom1 and exhibited luminal protrusions, a characteristic of activated endothelium (Fig. 3 E). In summary, the expression of prominin1 is quite widespread in the brain. In fact, we found Prom1 expression in cells as diverse as ependyma epithelial cells, a population of GFAP positive cells in the SVZ and SGZ and Prom1^+^ DCX neuroblasts. These neuroblasts are known to migrate and to become calretinin^+^ neurons in the olfactory bulb. In addition, non-neural cells, such as morphologically activated endothelial cells also express Prom1 in the brain.

### Stem-Cell like Properties of Prom1^+^ Cells

**Figure 4:**
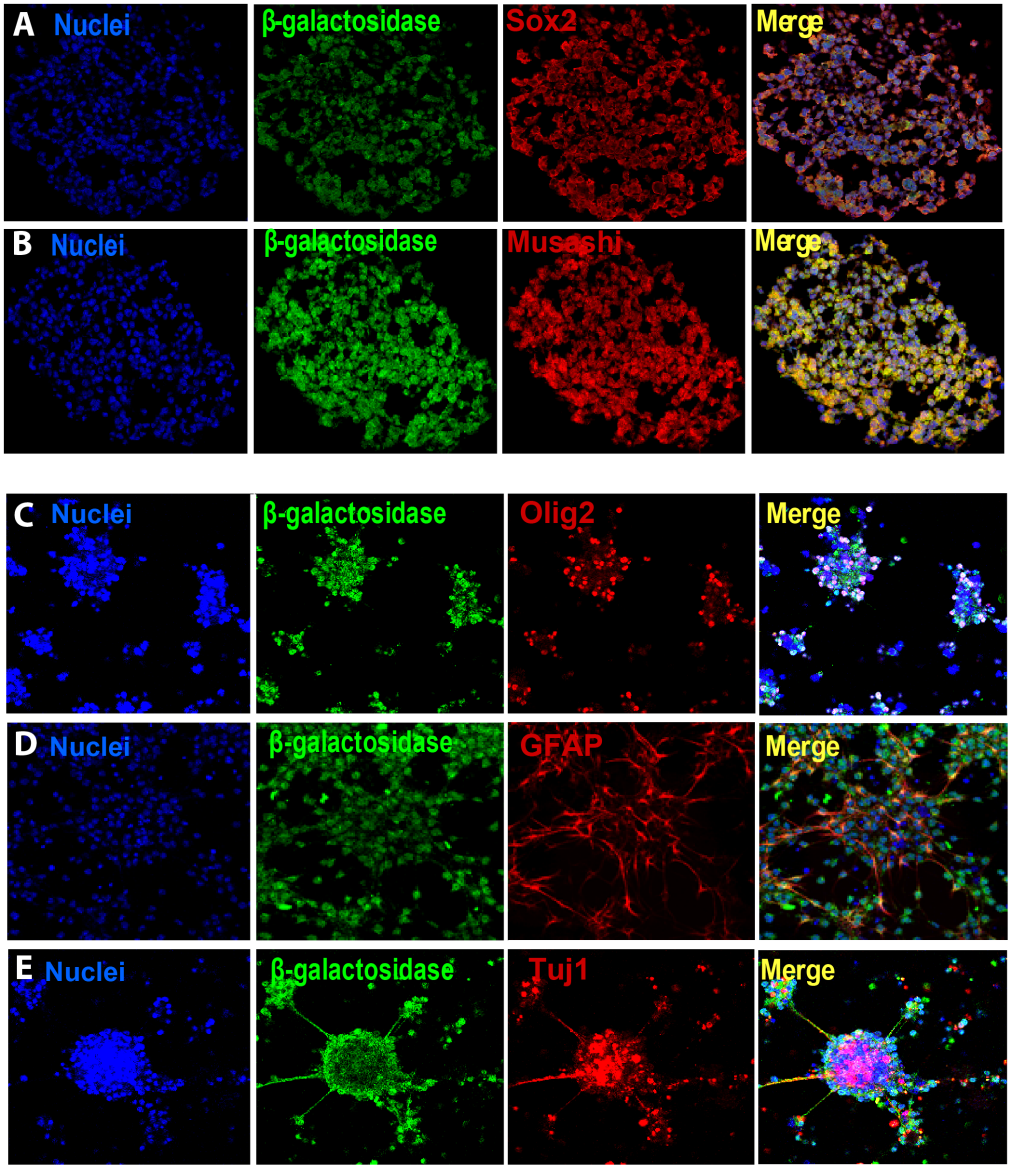
Isolated Prom1^+^ embryonic lateral ventricle and SVZ cells in culture give rise to neurospheres expressing neural cell markers but not vascular markers. Z-stack confocal images of cultured neurospheres generated from Prom1^+^ cells of E12.5 forebrain of prominin-1^lacZ/+^ mice in serum free media and in the presence of bFGF revealed co-immunostaining for ß-galactosidase (green) and both Sox2 (A) and Musashi (B) stem cell markers. Z-stack confocal images of secondary differentiated neurospheres induced by removing bFGF and plating on polyL-ornithine and fibronectin coated dishes from Prom1^+^ cells of E16.5–E17.5 forebrain of prominin-1^lacZ/+^ mice. Prom1^+^ cells (ß-galactosidase (green) in culture give rise to 3 different neural lineages (C) Olig 2 oligodendrocytes, (D) GFAP astrocytes and (E) Tuj1 neuron-specific progenitor neuronal marker.

To test for self-renewal and multipotency of Prom1^+^ cells, we generated neurosphere cultures from forebrains of *Prom1^lacZ/+^* mice after dissociation of SVZ and selection for Prom1^+^ and Prom1^−^ cells using anti-Prom1 microbeads. Both cell populations were cultured in serum free media in the presence of basic FGF (bFGF) and formed spheres. Multiple generations of spheres were produced after dissociation and culture of single cells. Co-immunostaining of undifferentiated neurospheres, sectioned and analyzed by confocal microscopy, showed that ß-galactosidase expressing cells co-stained for Sox-2 and Musashi-1 stem cell markers (Fig. 4 A, B). We then cultured the undifferentiated neurospheres after growth factor withdrawal to induce differentiation. Co-immunostaining of undifferentiated and differentiated neurospheres, derived from either Prom1^+^ or Prom1^−^ cells, for ß-galactosidase, GFAP, Olig 2 and Tuj1 (class III ß-tubulin), revealed the capacity of both Prom1^+^ and Prom1^−^ cells to generate all central nervous system lineage types (Fig. 4 C, D and E). To better quantify the relative contribution of Prom1^+^ and Prom1^−^ cells to neural specification, we used quantitative PCR (q-PCR) to measure the levels of *Sox2*, *Nestin*, *GFAP*, *olig2* and *Tuj1* in freshly isolated cells and cultured neurospheres. Our data suggests that *Sox2* and *Tuj 1* gene expression was slightly higher in Prom1^+^ cells than Prom1^−^ cells at day 0, while *GFAP* was lower. After 6 days in culture *GFAP* levels continued to decrease and were much lower in Prom1^+^ than in Prom1^−^ cells, although the difference was not statistically significant (Figure S3 panel A). In addition, Prom1^+^ isolated cells largely expressed VE-cadherin on day 0 and this expression dropped in culture overtime (Figure S3 panel A). This is corroborated by neurosphere generation from the forebrain of *Vegfr2-GFP* mice (Figure S3B and C). GFP is expressed when neurospheres are forming but is dramatically decreased in culture, supporting that only neurogenesis and gliogenesis occur under the culture conditions for neural cells.

### Prominin-1 is Expressed by Cells with Astrocyte and Endothelium Phenotypes in Brain Tumors

**Figure 5:**
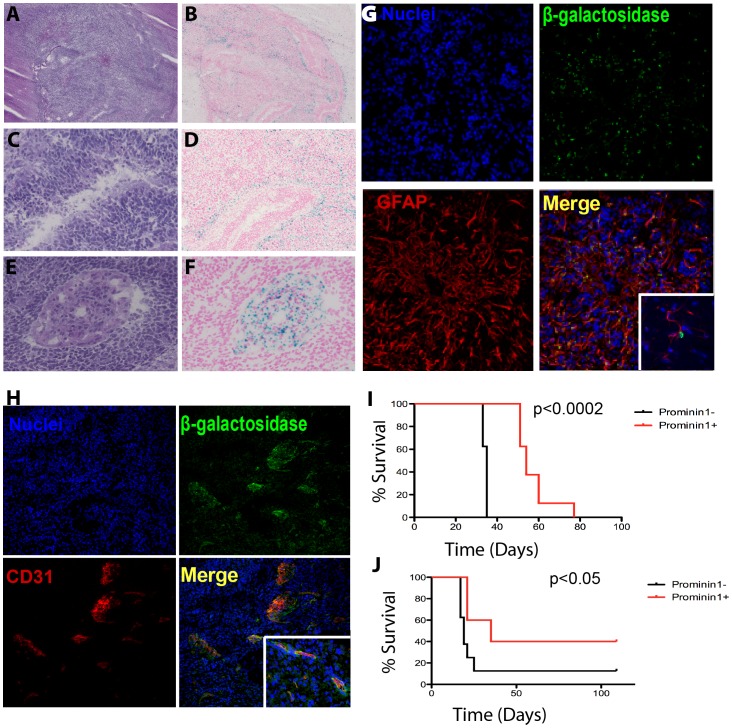
Prom1 is expressed by endothelial and tumor cells and both Prom1^+^ and Prom1^−^ cells contribute to glioma growth. H&E sections of brain tumor of 2 to 4 month old mouse generated by crossing *Prom1^lacZ/+^* and Ntv-a;*Ink4a-Arf^−/−^* mice infected with RCAS-PDGF. Tumors (A) were characterized by pseudopalisading necrosis (C) and microvascular proliferation (E), characteristic of glioblastoma. X-gal staining revealed ß-galactosidase activity throughout the tumor (B), at the periphery of pseudopalisading necrosis (D) and within microvascular proliferation“glomeruloid tuft” (F). (G) Z-stack and confocal optical sections revealed that some tumor cells coimmunostained for ß-galactosidase (green) and GFAP (red) and had astrocytic morphology (inset). (H) Other cells within the “glomeruloid tuft” coimmunostained for ß-galactosidase (green) and CD31 endothelial cells (red). (I) Kaplan-Meier analysis comparing survival of adult wild-type mice implanted with tumor cells dissociated from Prom1^+^ (N = 8) and Prom1^−^ neurospheres (N = 8) from PDGF-induced tumors in Ntv-a;*Ink4a-Arf^−/−^ Prom1^lacZ/+^* mice. (J) Survival curve of adult (8 weeks) wild-type mice implanted with Prom1^+^ (N = 5) and Prom1^−^ (N = 8) PDGF-induced tumors immediately after their cells were dissociated.

To determine the contribution of Prom1^+^ cells to the growth of the proneural GBM subtype, we generated Ntv-a;*Ink4a-Arf^−/−^ Prom1^lacZ/+^*mice and infected their brain with RCAS-PDGF. Histological analysis of tumors that formed in the brain of these mice stained with hematoxylin and eosin (H&E) revealed GBM-type of tumors (Fig. 5 A), with pseudopalisading necrosis (Fig. 5 C) and microvascular proliferation (Fig. 5 E). ß-galactosidase detection showed that some areas in the tumor showed high numbers of Prom1^+^ cells with other areas mostly devoid of them (Fig. 5 B). There appears to be a preferential distribution of Prom1^+^ cells in the tumor adjacent to the normal brain parenchyma (Fig. 5 B), in the periphery of pseudo-palisading necrosis (Fig. 5 D) and in areas of vascular hyperplasia (Fig. 5 F). To characterize Prom1^+^ brain tumor cells, we co-immunostained sections of the tumors with neural and vascular specific markers and anti-ß-galactosidase monoclonal antibodies. A subset of ß-galactosidase expressing cells manifested morphologic characteristics of large astrocytes and were GFAP^+^ (Fig. 5 G). Co-immunostaining of tumor sections with anti-ß-galactosidase and anti-CD31 antibodies revealed that a subset of Prom1^+^ cells in the tumors, were endothelial cells (Fig. 5 H). These cells were detected in areas of microvascular proliferation and glomeruloid tufts, in close proximity to the ventricles, and at the edge with brain parenchyma.

### Both Prominin1^+^ and Prominin1^−^ Proneural Tumor Cells are Capable of Tumor Initiation

To determine if prominin1^+^ cells are more efficient than Prom1^−^ in promoting tumor growth, we separated Prom1^+^ (24±4% of the total isolated cells) and Prom1^−^ tumor cells using anti- Prom1 microbeads and cultured them under stem cell conditions. The spheres formed were dissociated into single cell suspension and 20,000 cells were implanted into the brain of six to eight week old C57BL/6J mice. The incidence of tumors was 100% for mice implanted either with Prom1^+^ or Prom1^−^ cells and the induced tumors had characteristics of high-grade gliomas with areas of necrosis and pseudo-palisading and microvascular proliferation (Figure S4). Mice were sacrificed when they became symptomatic. Interestingly, survival was shorter for the mice implanted with Prom1^−^ negative cells (Fig. 5 I). To avoid *in vitro* selection bias by extended neurosphere culture, we implanted the same age C57BL/6J mice cells with Prom1^+^ and Prom1^−^ tumor cells (5,000 cells/mouse) immediately after anti-Prom1 microbead separation. Survival was again longer for the mice implanted with Prom1^+^ fraction (Figure 5J), suggesting that Prom1^+^ tumor cells are not essential for PDGF-driven proneural GBM growth.

### Prominin1^+^ Endothelial Cells Hasten Proneural Tumor Growth in the Brain

**Figure 6:**
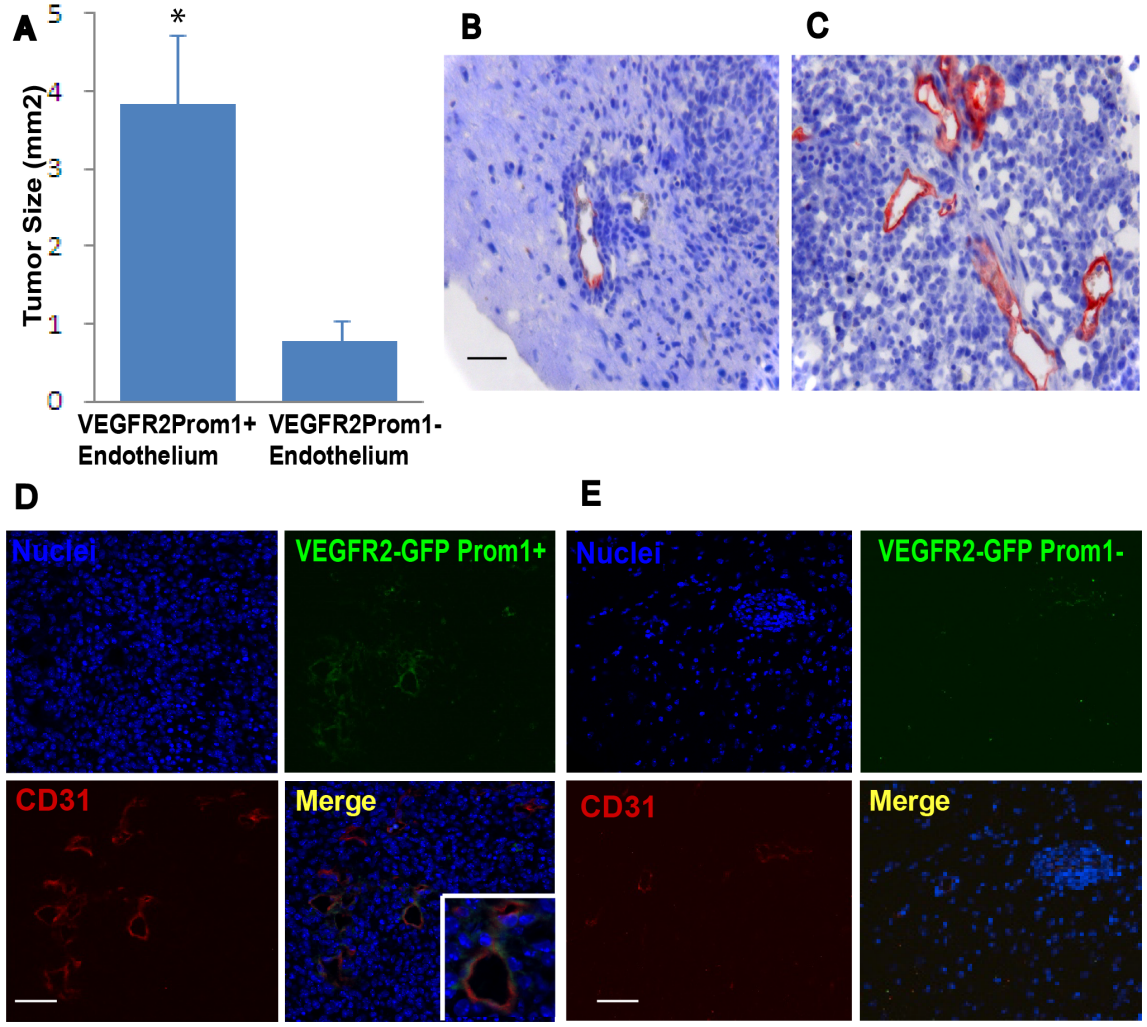
Tumors with endothelial cells expressing Prom1^+^ are larger and exhibit dynamic microvascular endothelial proliferation compared to tumors containing Prom1^−^ endothelium. (A) Stereotactic co-implantation into the adult mouse brain of cells dissociated from PDGF-induced tumorspheres in combination with sorted VEGFR2-GFP Prom1^+^ endothelial cells (N = 12) form tumors at four weeks after implantation with maximal cross-sectional area that is significantly larger than those produced by co-implantation with VEGFR2-GFP Prom1^−^ endothelium (N = 10). (B) Immunohistochemical detection of CD31 endothelial cells counterstained with hematoxylin confirmed paucity of CD31 endothelial cells in tumors implanted with VEGFR2-GFP Prom1^−^ endothelial cells, but (C) marked immunoreactivity in tumors implanted with VEGFR2-GFP Prom1^+^ endothelium. (D) Confocal images showing multiple areas of coimmunostaining for VEGFR2-GFP Prom1^+^ (green) and CD31 endothelial cells (red) (Inset, higher magnification). (E) Confocal images showing rare areas of coimmunostaining for VEGFR2-GFP Prom1^−^ (green) and CD31 (red) endothelial cells. Error bars depict the s.e.m. Scale bar, 50 µm (B, C) or 100 µm (D, E). *P<0.03.

To investigate whether Prom1^+^ endothelium is more important for proneural GBM subtype than Prom1^−^ endothelium, we used fluorescence-activated cell sorting (FACS) to isolate GFP^+^ Prom1^+^ and GFP^+^ Prom1^−^ endothelial cells from the brain of VEGFR2-GFP mice. We co-implanted six to eight week old C57BL/6J mice with dissociated cells from tv-a;*Ink4a-Arf^−/−^* tumorspheres and VEGFR2-GFP Prom1^+^ or VEGFR2-GFP Prom1^−^ endothelium (Fig. 6). Mice were sacrificed at four weeks after tumor implantation and the maximal cross-sectional area of the tumors was measured on coronal sections. VEGFR2-GFP Prom1^+^ endothelium induced tumors had significantly increased size compared to tumors that contained VEGFR2-GFP Prom1^−^ endothelium (P<0.03) (Fig. 6A). The increased tumor burden created by Prom1^+^ endothelium suggests its function in fostering tumor growth.

To investigate the mechanistic role of Prom1^+^ endothelium in promoting growth of PDGF-enriched proneural GBM, we first analyzed the vascular density by quantifying the number of vessels present throughout the tumors. The mean number of vessels per high power field within the GFP^+^ Prom1^+^ tumors was 36.6±9.8 vessels and it was only 6.8±1.3 vessels in the tumors with GFP^+^ Prom1^−^ endothelium (P<0.0001). Tumors with GFP^+^ Prom1^+^ endothelium had larger, more distorted vessels with thickened walls and exhibited increased tumor cell density (Fig. 6 B and C). The transplanted Prom1^+^ endothelium expressed CD31 after engraftment, in contrast to the Prom1^−^ endothelium, indicating that the transplanted Prom1^+^ endothelium engrafts retained their endothelial identity (Fig. 6 D and E). The significantly increased vessel density in the tumor clearly indicated that Prom1^+^ endothelium is capable of incorporating into the proneural glioma vasculature, triggering a higher vasculogenic activity to establish functional tumor vessels.

It has been recently suggested that endothelium not only serves as a passive conduit to deliver oxygen to tumors, but also exerts paracrine (angiocrine) factors that stimulate tumor growth. To identify potential angiocrine factors produced by Prom1^+^ endothelium and responsible for the difference in tumor growth and characteristics, we analyzed FACS-isolated normal CD31^+^ Prom1^+^ and CD31^+^ Prom1^−^ vasculature by Affymetrix microarray (Table 1 and 2). We observed an upregulation of endothelial cell-specific genes in Prom1^+^ endothelium, including lipocalin 2, selectin, endothelin 1 and 3, nestin, VEGF C, and nitric oxide synthetase 3, suggesting that Prom1^+^ endothelium is the producer of a set of active angiocrine factors contributing to the growth of proneural GBM subtype. The differential expression of some of these factors between Prom1^+^ and Prom1^−^ endothelial cells was validated by quantitative PCR (Figure S5). One of those factors, nitric oxide, has been shown to promote tumor initiation and stem cell-like characteristics in glioma [22]. Taken together, Prom1^+^ endothelium fosters proneural tumor growth, not only by establishing perfused vessels to meet the metabolic needs, but also by relaying stimulatory angiocrine factors.

**Table 1:** Gene Expression Differences between Prom1^+^ and Prom1^−^ Endothelium (upregulated).

Gene Identifier	Fold Increase	Symbol	Product
1427747_a_at	694.5	Lcn2	lipocalin 2
1417263_at	59.22	Ptgs2	prostaglandin-endoperoxide synthase 2
1420558_at	40.93	Selp	selectin, platelet
1421712_at	21.66	Sele	selectin
1417932_at	3.13	Il18	interleukin 18
1429084_at	2.97	Vezf1	vascular endothelial zinc finger 1
1420653_at	2.91	Tgfb1	transforming growth factor, beta 1
1451314_a_at	2.91	Vcam1	vascular cell adhesion molecule 1
1425656_a_at	2.88	Baiap2	brain-specific angiogenesis inhibitor 1-associated protein 2
1441923_s_at	2.75	Edn3	endothelin 3
1434651_a_at	2.54	Cldn3	claudin 3
1439766_x_at	2.51	Vegfc	vascular endothelial growth factor C
1418547_at	2.51	Tfpi2	tissue factor pathway inhibitor 2
1449022_at	2.46	Nes	nestin
1420664_s_at	2.45	Procr	protein C receptor, endothelial
1451924_a_at	2.25	Edn1	endothelin 1
1432181_s_at	2.15	Ecgf1	endothelial cell growth factor 1 (platelet-derived)
1434092_at	2.12	Nos3	nitric oxide synthase 3, endothelial cell
1451024_at	2.04	Edg6	endothelial differentiation, G-protein-coupled receptor 6

Functionally relevant genes upregulated in prom1^+^ endothelial cells compared to prom1^−^ endothelial cells.

**Table 2:** Gene Expression Differences between Prom1^+^ and Prom1^−^ Endothelium (downregulated).

Gene Identifier	Fold Decrease	Symbol	Product
1451691_at	0.0745	Ednra	endothelin receptor type A
1449351_s_at	0.0831	Pdgfc	platelet-derived growth factor, C polypeptide
1448606_at	0.12	Edg2	endothelial differentiation, lysophosphatidic acid G-protein-coupled receptor, 2
1437173_at	0.245	Edg3	endothelial differentiation, sphingolipid G-protein-coupled receptor, 3
1450297_at	0.368	Il6	interleukin 6
1421106_at	0.427	Jag1	jagged 1

Functionally relevant genes downregulated in prom1^+^ endothelial cells compared to prom1^−^ endothelial cells.

## Discussion

The currently accepted hypothesis is that rare specific cells give rise to and maintain a number of malignancies including brain tumors [4], [5]. These cells are characterized by the expression of CD133, however the existence of these brain tumor stem cells has been a matter of controversy because of conflicting supporting evidence. While some studies have attributed tumor initiation to the CD133^+^ brain tumor cells [4], [5], others have shown that CD133^−^ cells can also have stem cell-like properties and potentially drive GBM tumors [23]. It is also known that gliomas are heterogeneous tumors with different molecular abnormalities and, in GBM, different molecular subclasses have been identified [1], [2], [3]. Thus, it is conceivable that the tumor cell of origin may not be the same for each subclass of GBM, and therefore, CD133^+^ brain tumor cells may differentially influence each of these subclasses. To study the role of CD133 in the proneural GBM subclass we used a PDGF-driven *Prom1^lacZ/+^*reporter mouse model. We found no increase in glioma initiation by implantation of Prom1^+^ dissociated cells from Prom1^+^ over Prom1^−^ tumorsphere cells, suggesting no advantage of Prom1^+^ cells in the growth of proneural brain tumor enriched in PDGF signaling.

In an orthotopic model, brain-tumor stem-like cells are maintained in perivascular niches also containing endothelial cells. The vasculature hastens tumor growth, not only by delivering nutrients and oxygen but also by release of paracrine factors [24]. The expression of Prom1 by a subset of endothelial cells in the brain does not occur outside the nervous system (unpublished observation). When we implanted Prom1^+^ or Prom1^−^ cells from PDGF-driven proneural tumors immediately after cell dissociation, no difference in growth between Prom1^+^ and Prom1^−^ tumors was observed. However, when dissociated cells from proneural tumor were co-implanted with Prom1^+^ endothelium isolated from normal brain we found that Prom1^+^ endothelium was crucial to promote tumor growth.

Furthermore, based on our microarray and quantitative PCR results, the Prom1^+^ fraction of brain endothelial cells expressed genes coding for trophogens, endothelial-specific adhesion molecules and endothelial-derived chemokines that are associated with recruitment of inflammatory cells, tumor angiogenesis and growth [25]. For instance, endothelial nitric oxide synthetase is responsible for the production of nitric oxide and has been linked to the acceleration of tumor growth in PDGF-driven gliomas [22]; lipocalin-2 or neutrophil gelatinase-associated lipocalin forms a complex with MMP-9 and protects it from degradation, facilitating tumor invasion [26], [27]; Vascular-derived endothelin 3 (Edn3) has been shown to direct axonal growth and guidance in the peripheral nervous system [28], and endothelin-1 (Edn1), whose vasoconstrictive effects can be terminated by stimuli from sensory-motor nerves [29], has also been associated with tumorigenesis [25]. In contrast, our results demonstrate that the inflammatory and activated endothelium properties observed in the Prom1^+^ endothelium were downregulated in the Prom1^−^ endothelium. Electron microscopy images showed that Prom1^+^ endothelium displayed luminal protrusions, a characteristic of activated endothelium. In our tumors, Prom1 was not only expressed by endothelium but also by large GFAP^+^ tumor cells. We showed that these cells reside in close proximity to the vasculature and immediately outside of pseudopalisading necrosis, an area known for severe hypoxia. Hypoxia increases the levels of CD133 expression and oxygen tension can influence posttranslational modification of CD133 [30]. Hypoxia is a potent promoter of neo-angiogenesis, enhancing invasion [31], [32] and forcing differentiation of glioma cells into endothelial-like cells [33]. This transdifferentiation of tumor cells into endothelial-like cells may occur through a VEGF-independent pathway. It is possible that there are multiple alternate pathways of resistance to anti-VEGF treatment that may depend on other angiocrine factors produced by tumor endothelial cells [34] and we believe we have identified some of those factors that are relevant for proneural brain tumor model.

The adult SVZ is known to give rise to transient-amplifying neural progenitors (C-cells) that generate DCX^+^ neuroblasts (A-cells) that migrate and differentiate into interneurons in the olfactory bulb [35], [36]. Current evidence suggests that the adult neurogenic niches that contain neural stem cells are organized as pinwheels with the apical portion of the neural stem cell (B_1_ cell) at the core, in the lateral ventricle surface, ependymal cells at the periphery and the basal portion of the B_1_ cell contacting a blood vessel [37] surrounded by a complex vascular plexus in the SVZ [14], [15]. Neural stem cells in the SVZ and in the SGZ of the hippocampus have morphological characteristics of astrocytes and are GFAP^+^ (B cells) [38]. We show in this study that ependyma, neural progenitor cells, RMS neuroblasts and olfactory bulb calretinin interneurons express Prom1.

There has been some recent controversy on whether or not adult ependyma cells are a source of neural stem cells [37], [39]. Our data demonstrate that Prom1^+^ cells that reside in the ependyma have no self-renewal properties and do not generate neural precursors. However, in the SVZ and SGZ we identified GFAP^+^ Prom1^+^ cells with morphological characteristics of large stem-cell like astrocytes. It has been shown that GFAP^+^ astrocytes (B cells) also generate oligodendrocytes [40]. In fact, we did find Prom1^+^ cells co-expressing Olig2 in the SVZ and SGZ, but these were rare. We also found Prom1^+^ Purkinje cells, and even though we have not isolated Prom1^+^ cells from cerebellum and explored their stem cell properties, CD133^+^ expressing cells have been identified in the postnatal cerebellum including the Purkinje cell layer [41]. The expression of Prom1 by a variety of cells in the brain with polarized morphology, such as SVZ and SGZ astrocytes, Purkinje cells and activated endothelial cells may indicate that prom1 is a characteristic of specialized epithelial cells.

Together our data showing expression of Prom1 in neural and endothelial cells reveals a new relationship within the neurovascular niche involving Prom1 in angiogenesis and the progression of proneural tumors. Further work is needed to determine whether selective targeting of angiocrine factors in brain Prom1^+^ endothelial cells will block glioma growth in proneural GBM tumors and whether Prom1^+^ endothelial cells are determinant in sustaining tumor growth in other GBM subclasses.

## Materials and Methods

### Ethics Statement

All animal studies were performed in accordance with protocols approved by Institutional Animal Care and Use Committee of Memorial Sloan-Kettering Cancer Center and Weill Cornell medical College and in accordance to National Institute of Health guidelines for animal welfare (IACUC permit 06-04-005).

### Transgenic Reporters, Brain Tumor Mouse Model and Mouse Surgery

C57BL/6J mice were obtained from Jackson Laboratories (Bar Harbor, ME). *Prom1^lacZ/+^* mice and Tva;*Ink4a-ARF^−/−^* were a gift from Dr. Shahin Rafii and Dr. Eric Holland (Memorial Sloan-Kettering Cancer Center, NY), respectively [12], [42]. *VEGFR2-GFP* mice were acquired from Dr. Janet Rossant (the Hospital for Sick Children, Canada). To generate tumors in the Tva;*Ink4a-ARF^−/−^* mice, 1 µl of approximately 10^4^ DF-1 cells expressing RCAS-PDGF were injected intracranially within 72 hrs of birth using a Hamilton syringe. For co-implantation of VEGFR2-GFP endothelial cells, C57BL/6J mice were anesthetized with 100 mg/kg intraperitoneal ketamine and 10 mg/kg xylazine, intraperitoneal. Injections were performed using stereotactic frame (Stoelting, Wood Dale) at the following coordinates, anterior 1.7 mm, lateral 0.5 mm and depth 2.5 mm. Mice were monitored daily for symptoms of tumor development, such as withdrawal from activity, seizures, weakness, head tilt and weight loss. Animals presenting with any signs of morbidity were promptly euthanized by exposure to100% carbon dioxide in accordance with IACUC approved protocol.

### Cells and Tissue Samples

Brain tumor and normal brain tissue were dissected in Ca^2+^ -Mg^2+^ -free HBSS, minced into PIPES solution, and then digested in papain-PIPES (Worthington Biochemical) and DNase I at 37°C. Cells were collected by centrifugation at 200 g and re-suspended in Dulbecco’s minimum essential medium (DMEM)/F12, followed by mechanical dissociation with fire-polished Pasteur pipettes. Cells were passed through 40 µm mesh into DMEM/F12 with 10% plasma-derived fetal bovine serum (PD-FBS; Cocalico Biologicals) to stop enzymatic dissociation [43]. Cells were spun down and re-suspended in PBS supplemented with 1% bovine serum albumin (BSA) and 2 mM EDTA and incubated in anti-prominin-1 microbeads (magnetic –activated cell sorting [MACS]; Miltenyi Biotech) according to manufacturer’s. Embryonic forebrain ventricular, subventricular and cells from tumors were expanded in serum-free NeuroCult Neural Stem cell basal medium, supplemented with NeuroCult proliferation supplements (Stem Cell Technologies) with 20 ng/ml basic fibroblast growth factor (bFGF) (Invitrogen). bFGF was removed from the medium and cells were plated on poly-L-ornithine and fibronectin coated culture dishes for cell differentiation.

DF-1 cells were purchased from ATCC. Cells were grown at 39°C according to ATCC instructions and transfected with RCAS-PDGF by using Fugene 6 transfection kit (Roche #11814443001). Transfections were checked by Western lot and PCR.

### ß-galactosidase Detection, Immunohistochemistry (IHC) and Immunofluorescence

Brains of 2–4 month old mice were frozen in OCT Compound (Sakura Finetek, CA) for IHC. Cryosections (10 µm) were fixed in ice-cold 4% paraformaldehyde (PFA) for 10 minutes and then rinsed with PBS three times. After endogenous peroxidase and nonspecific protein block (5% BSA, 10% donkey serum, and 0.02% Tween-20), CD31 clone Mec 13.3 (1∶100;BD Biosciences 553370 Pharmigen) was incubated overnight at 4°C. After secondary pAb and streptavidin horseradish peroxidase incubations (Jackson ImmunoResearch, PA), staining was developed with AEC^+^ (DAKO) and briefly counterstained in Mayer's hematoxylin (DAKO). For ß-galactosidase detection, sections of fresh-frozen tissues of *Prom1^lacZ/+^* mice were incubated with X-gal (Calbiochem at 37°C for 12–16 hr and counterstained with Nuclear Fast Red (Vector Laboratories).

For electron microscopy, mice were perfused with 4% PFA and 2.5% glutaraldehyde, followed by X-gal staining of 1 mm^3^ selected areas of the brain. For immunofluorescence, mice were perfused with PBS and 4% PFA. Forty micron free-floating sections were post-fixed with 4% PFA. Sections were blocked (5% normal horse and goat serum (Jackson ImmunoResearch, PA)/0.1% Triton X-100) and incubated in primary Abs: rabbit anti-ß-galactosidase polyclonal Ab (1∶1000;Chemicon), anti-ß-galactosidase mAb (1∶500;Promega), anti-glial fibrillary acid protein (GFAP) polyclonal Ab (1∶4000; Dako), neuronal Class III ß-tubulin mAb (Tuj1) (1∶500;Covance), NeuN mAb (1∶1000;Chemicon), anti-Olig2 polyclonal (1∶1000; Chemicon), CD31 clone Mec 13.3 mAb (1∶100; BD Biosciences Pharmigen), anti-SMI-91R (myelin CNPase) mAb (1∶200; Covance), Musashi-1 14H-1 ascitis (1∶100 gift from Dr. Hideyuki Okano, Keio University Japan), anti-mouse Calbindin polyclonal (1∶500;abcam), anti-calretinin polyclonal (1∶1000; Swant), nestin (1∶50;BDBioscience), Sox 2 polyclonal Ab (1∶200; Neuromics), DXC polyclonal Ab (1∶500; Abcam). After incubation in fluorophore-conjugated secondary antibodies (2.5 µg/ml, Jackson ImmunoResearch, PA), sections were counterstained with TOPRO3 or DAPI (Invitrogen, CA).

### Image Acquisition, Image Analysis, Flow Cytometric Analysis and Sorting

Bright-field micrographs were captured on an Olympus microscope using AxioCam (Zeiss). For electron microscopy, sections were processed in 1% osmium and 1.5% ferricyanide, stained in 1.5% Uranyl acetate, embedding in EMbed resin (Electron Microscopy Sciences, Hatfield). Sections were cut at 55–60 nm on an RMC MT-7000 Ultramicrotome (Boeckeler-RMC Instruments, Tucson, AZ) and contrasted with lead citrate. For flow cytometry, cells were incubated with phytoerythrin (PE) anti-mouse Prom1 and CD31 PE Cy7 (eBioscience) and analyzed on LSRII-SORP (BD Biosciences). Doublets were excluded by FSC-W × FSC-H and SSC-W × SSC-H analysis, and single stained channels were used for compensation. For FACS, cells were incubated with CD31 PE Cy7 and phytoerythrin (PE) anti-mouse Prom1 (eBioscience) and two-channels were used to define GFP^+^ Prom1^+^ and GFP^+^ Prom1^−^ subpopulations and CD31^+^ Prom1^+^ and CD31^+^ Prom1^−^ subpopulations. Sort gates were set in bivariate dot plots of the GFP and PE channels, and the fluorochromes were excited by 488 nm blue laser light.

### Quantitative Real-time PCR Analysis and Affymetrix Analysis

Total RNA was isolated from brain cells, neurosphere cultures or immediately after sorting, using RNeasy mini kit (Qiagen) and converted to cDNA using Superscript II Reverse Transcriptase (Invitrogen, CA) according to manufacturer’s instructions. The quantity and quality of the total RNA was assessed by using NanoDrop ND-1000 Spectrophotometer (Thermo Scientific) and Agilent Bioanalyzer. Quantitative PCR was carried out using SYBR Green expression systems (Applied Byosystems) and gene-specific primers (Table S1). For microarray profiling, RNA samples were amplified and labeled with biotin using the Ovation Biotin system (NuGEN Technologies, CA). The biotin labeled cDNA was fragmented and hybridized to the Mouse Genome 430 Plus 2.0 GeneChip arrays (Affymetrix, CA). GeneChip arrays were washed, stained, and scanned by GeneChip Scanner 3000 7G according to the Affymetrix Expression Analysis technical Manual and images acquired using Affymetrix GeneChip Operating Software. The target signal intensity from each chip was scaled to 500. The data normalization and analysis was performed with GeneSpring software (Agilent, Redwood City, CA). Gene Ontology analysis was performed using Pathway Express software and using annotation available from Kyoto Encyclopedia of Genes and Genomes (KEGG) pathways (http://www.genome.jp/kegg). The microarray gene expression data files were submitted to NCBI Gene Expression Omnibus (GEO) under accession number GSE43931.

### Statistics

Kaplan-Meier survival curves were generated with Prisma software. Analysis of cross-sectional area of the tumors was performed using a two-tailed Student’s t-test.

## Supporting Information

Figure S1 **X-gal staining detects Prom1^+^ cells from the ependyma to the olfactory bulb of the brain of an eight-week old** ***Prom1^lacZ/+^*** **mouse model.** (a and c, left panel) Low-power and respective magnified images (b and d, right panel) of anteroposterior coronal sections showing the distribution of ß-galactosidase activity in the olfactory bulb (a and b), and corpus callosum and ependyma (c and d). Abbreviations: ac, anterior commissural; aci, anterior commissure intrabulbar; cc, corpus callosum; Ep, ependyma; Epl, external plexiform layer olfactory bulb; Ipl, internal plexiform layer olfactory bulb; LSn, lateral septus nucleus; LV, lateral ventricle; MC, motor cortex; Mi, mitral layer olfactory bulb; Msn, medial septal nucleus.
(TIFF)

Figure S2 **Prom1^+^ cells are detected in the forebrain of** ***Prom1^lacZ/+^*** **adult mice.** Z-stack confocal images revealed coimmunostaining for ß-galactosidase (green) and calretinin (red) neurons in the olfactory bulb (A), doublecortin neuroblasts (red) in the SVZ and in RMS (B), GFAP (red) large astrocytic cells in the SVZ area (C), CD24 (red) ependymal cells (D) and BrdU (red) labeled cells in the SVZ (E). (F) No co-immunostaining was detected three weeks after BrdU administration. (G) No coimmunostaining was found for ß-galactosidase (green) and BrdU cells (red) in the ependyma. (H) Electron micrograph showed electron-dense X-gal crystals in the cytoplasm of ependymal cells and (I) nucleus and nuclear membrane of ependymal cell. (Insets B, C and D) higher magnification of confocal optical sections. Ep, ependyma; LV, lateral ventricle; m, mitochondria; N, nucleus; Arrows point to X-gal crystals and arrowheads to X-gal crystals in the nucleus.
(TIFF)

Figure S3 **(A) Relative quantification of gene expression levels determined by qPCR of Prom1^+^ and Prom1^−^ cells at day 0 and after 6 days in culture under stem cell conditions.** In each graph the level of expression of Prom1^−^ cells is set to 100% and is not shown. The relative amount of expression in Prom1^+^ versus is Prom1^−^ is shown in percentage. Confocal images of culture of embryonic brain of VEGR2-GFP mouse under stem cell conditions showed (B) expression of GFP (green) after two days in culture and (C) no expression of VEGR2-GFP after four days in culture.
(TIFF)

Figure S4 **Characteristics of tumor derived from Prom1^+^ cells and Prom1** ^−^ **cells. (**A) H&E section of tumor derived from Prom1^+^ neurospheres revealed pseudopalisading necrosis (pp) and microvascular proliferation “glomeruloid tuft” (gt). H&E section of tumors derived from Prom1^−^ neurospheres included (B) pseudopalisading necrosis and (C) microvascular hyperplasia characteristics of high-grade glioma.
(TIFF)

Figure S5 **qPCR validation of selected genes from Prom1^+^ endothelial cells.**
(TIFF)

Table S1 **List of primers used for qPCR.**
(DOCX)
