# Supplementary figures and images for: Prominin 1/CD133 Endothelium Sustains Growth of Proneural Glioma
Source: PLoS One. 2013 Apr 25;8(4):e62150. doi: 10.1371/journal.pone.0062150 (PMC3636202; doi:10.1371/journal.pone.0062150)

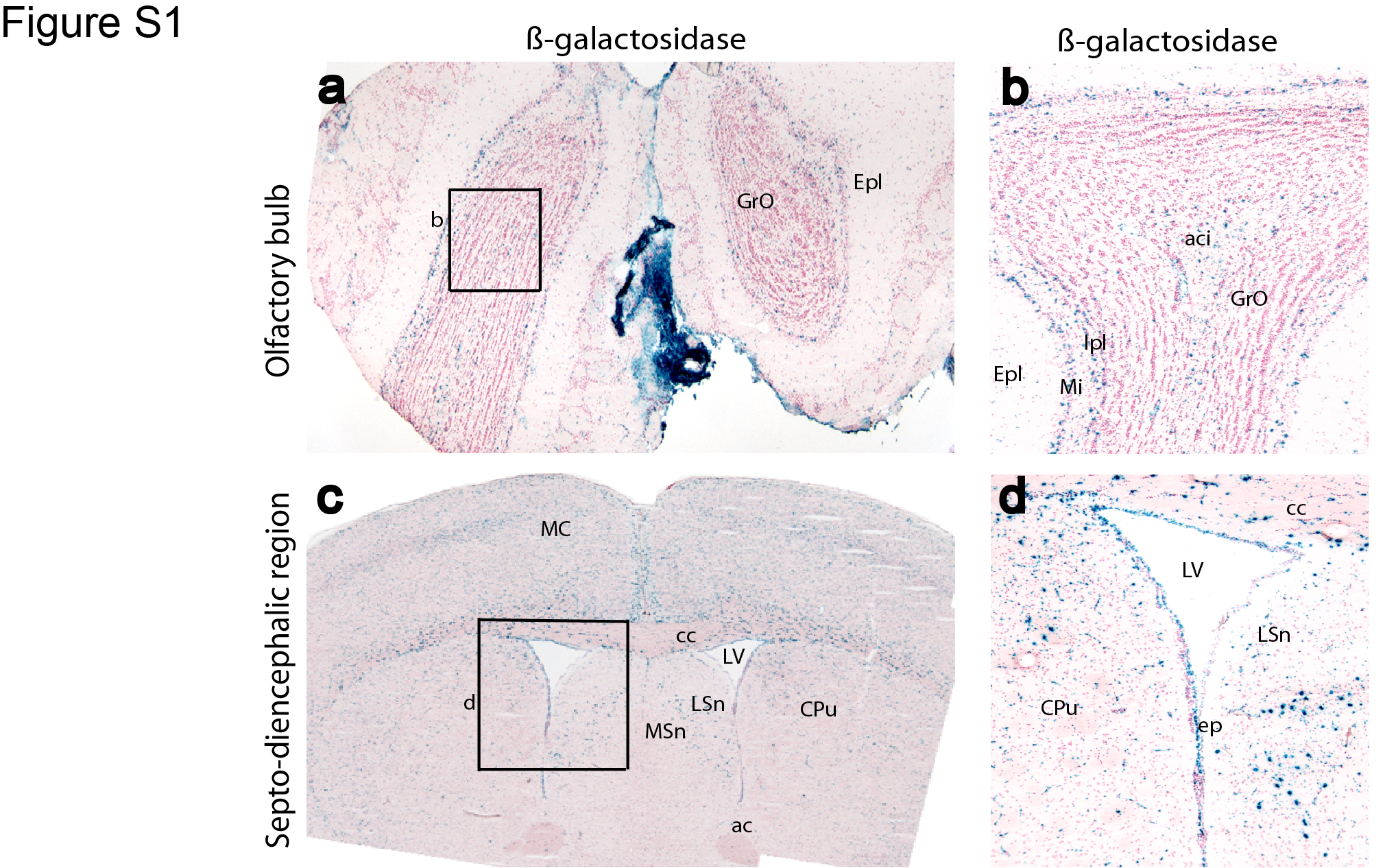

Supplement: Figure S1 — X-gal staining detects Prom1+ cells from the ependyma to the olfactory bulb of the brain of an eight-week old Prom1lacZ/+ mouse model. (a and c, left panel) Low-power and respective magnified images (b and d, right panel) of anteroposterior coronal sections showing the distribution of ß-galactosidase activity in the olfactory bulb (a and b), and corpus callosum and ependyma (c and d). Abbreviations: ac, anterior commissural; aci, anterior commissure intrabulbar; cc, corpus callosum; Ep, ependyma; Epl, external plexiform layer olfactory bulb; Ipl, internal plexiform layer olfactory bulb; LSn, lateral septus nucleus; LV, lateral ventricle; MC, motor cortex; Mi, mitral layer olfactory bulb; Msn, medial septal nucleus. (TIFF) [file pone.0062150.s001.tiff]

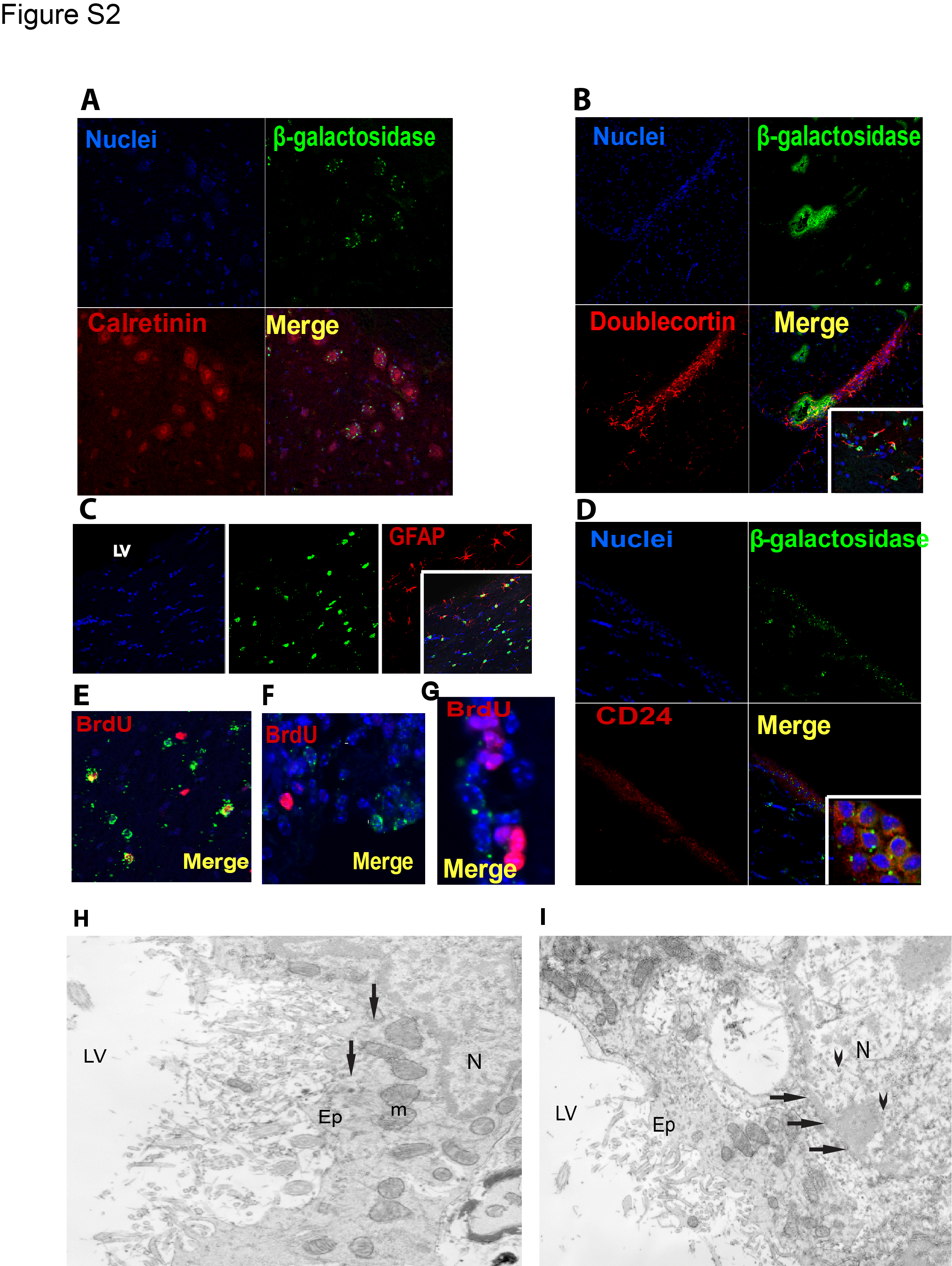

Supplement: Figure S2 — Prom1+ cells are detected in the forebrain of Prom1lacZ/+ adult mice. Z-stack confocal images revealed coimmunostaining for ß-galactosidase (green) and calretinin (red) neurons in the olfactory bulb (A), doublecortin neuroblasts (red) in the SVZ and in RMS (B), GFAP (red) large astrocytic cells in the SVZ area (C), CD24 (red) ependymal cells (D) and BrdU (red) labeled cells in the SVZ (E). (F) No co-immunostaining was detected three weeks after BrdU administration. (G) No coimmunostaining was found for ß-galactosidase (green) and BrdU cells (red) in the ependyma. (H) Electron micrograph showed electron-dense X-gal crystals in the cytoplasm of ependymal cells and (I) nucleus and nuclear membrane of ependymal cell. (Insets B, C and D) higher magnification of confocal optical sections. Ep, ependyma; LV, lateral ventricle; m, mitochondria; N, nucleus; Arrows point to X-gal crystals and arrowheads to X-gal crystals in the nucleus. (TIFF) [file pone.0062150.s002.tiff]

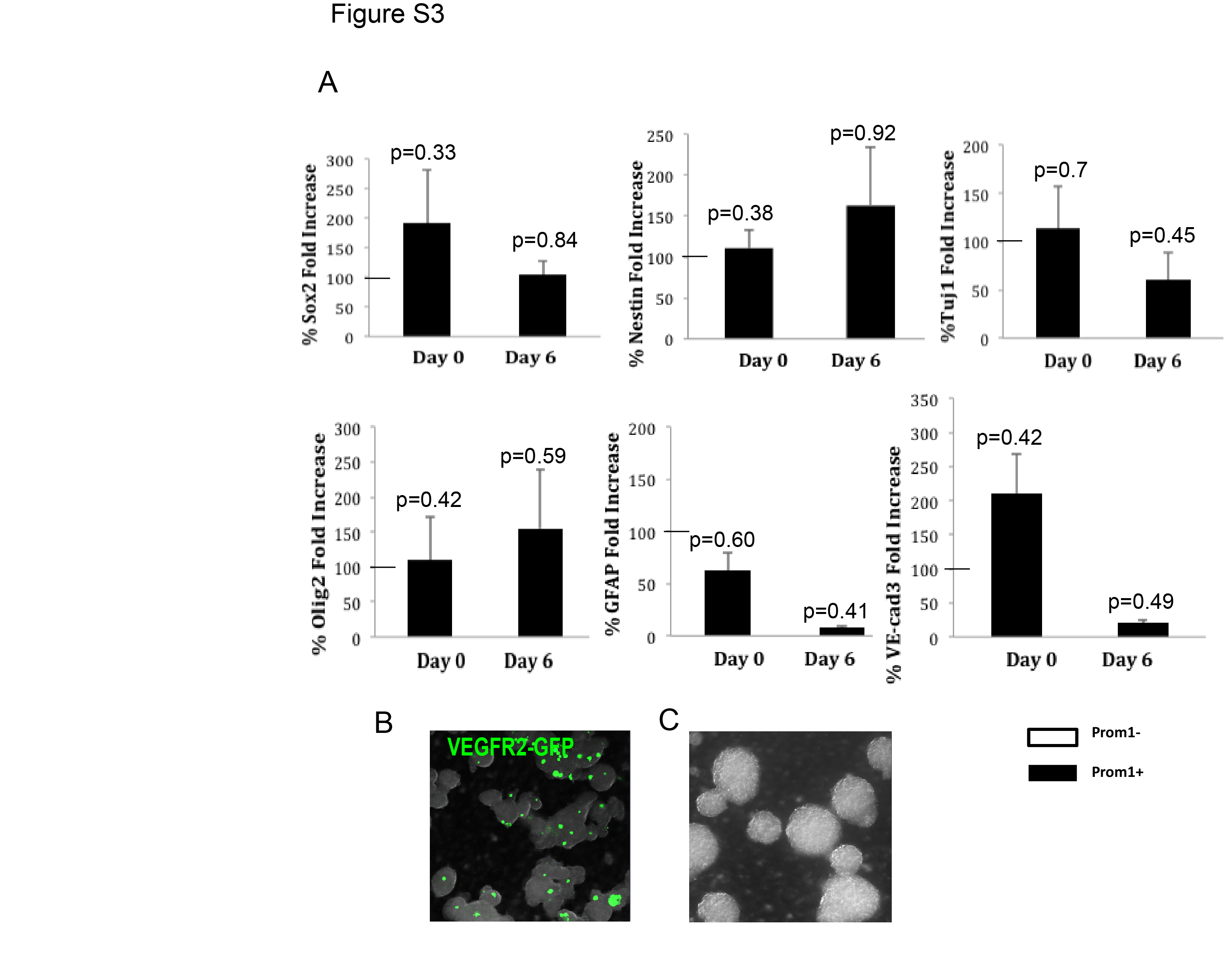

Supplement: Figure S3 — (A) Relative quantification of gene expression levels determined by qPCR of Prom1+ and Prom1− cells at day 0 and after 6 days in culture under stem cell conditions. In each graph the level of expression of Prom1− cells is set to 100% and is not shown. The relative amount of expression in Prom1+ versus is Prom1− is shown in percentage. Confocal images of culture of embryonic brain of VEGR2-GFP mouse under stem cell conditions showed (B) expression of GFP (green) after two days in culture and (C) no expression of VEGR2-GFP after four days in culture. (TIFF) [file pone.0062150.s003.tiff]

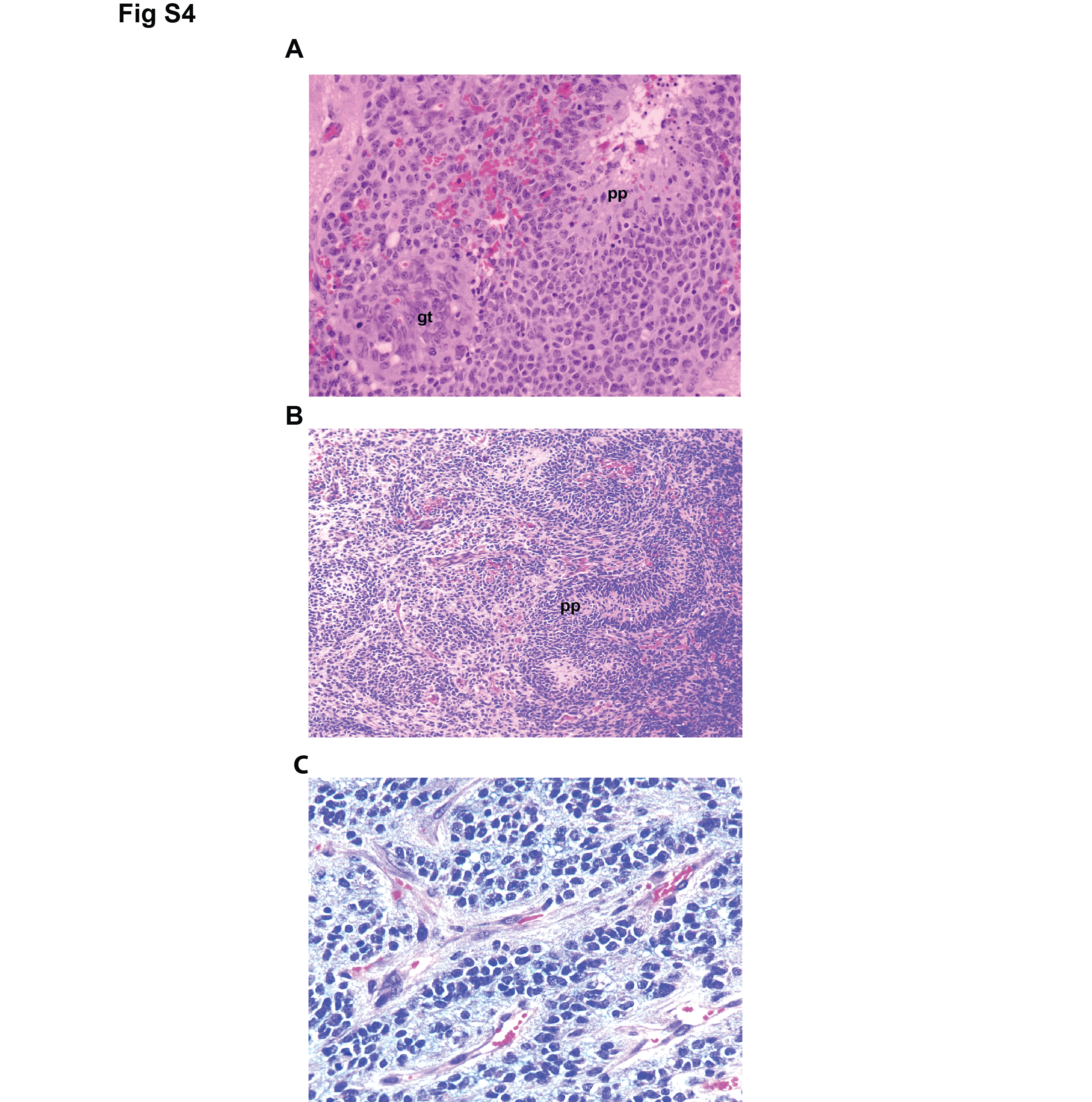

Supplement: Figure S4 — Characteristics of tumor derived from Prom1+ cells and Prom1 − cells. (A) H&E section of tumor derived from Prom1+ neurospheres revealed pseudopalisading necrosis (pp) and microvascular proliferation “glomeruloid tuft” (gt). H&E section of tumors derived from Prom1− neurospheres included (B) pseudopalisading necrosis and (C) microvascular hyperplasia characteristics of high-grade glioma. (TIFF) [file pone.0062150.s004.tiff]

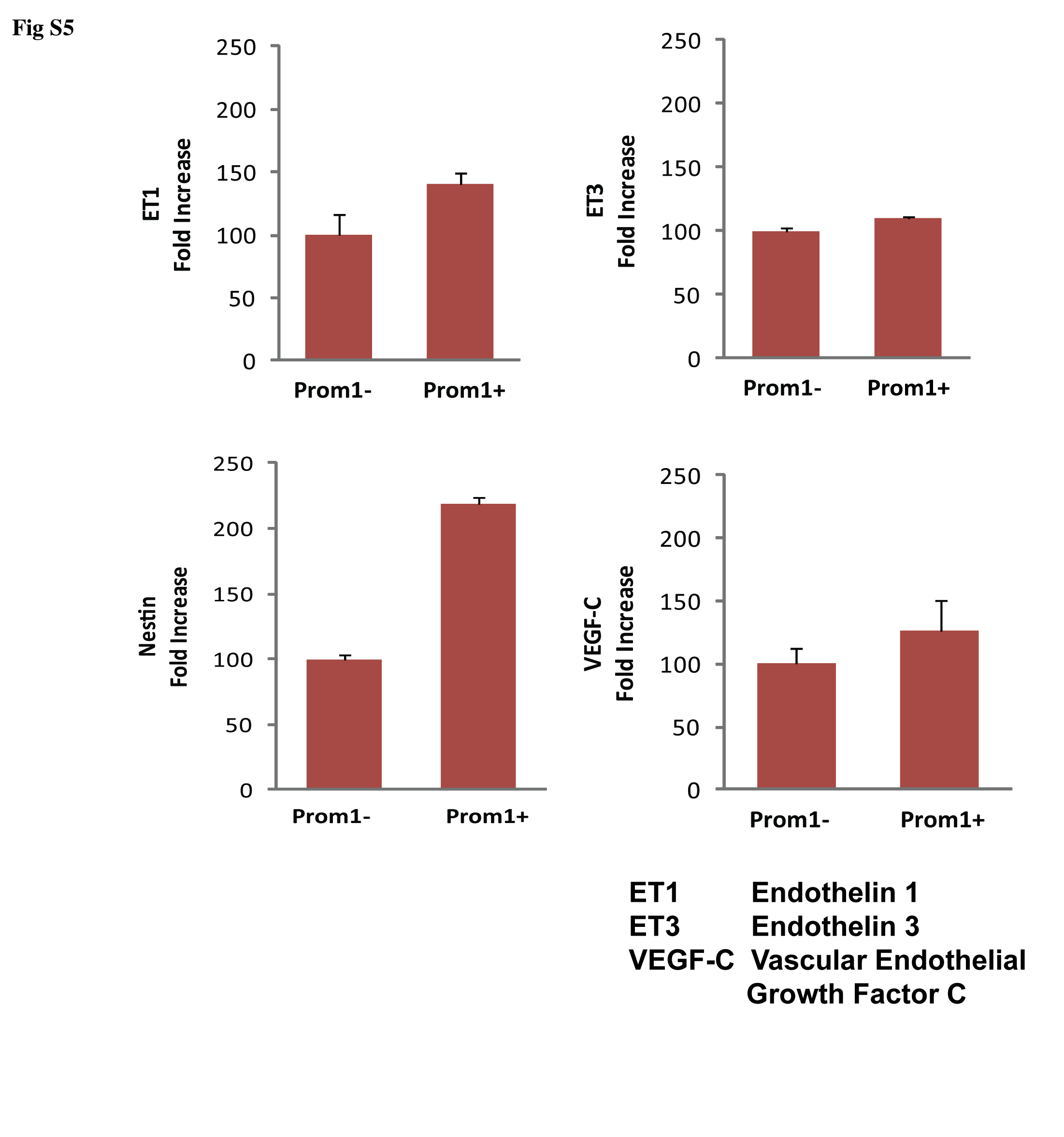

Supplement: Figure S5 — qPCR validation of selected genes from Prom1+ endothelial cells. (TIFF) [file pone.0062150.s005.tiff]
