# Supplementary material for: Prominin 1/CD133 Endothelium Sustains Growth of Proneural Glioma
Source: PLoS One. 2013 Apr 25;8(4):e62150. doi: 10.1371/journal.pone.0062150 (PMC3636202; doi:10.1371/journal.pone.0062150)
Supplement: Table S1 — List of primers used for qPCR. (DOCX) [file pone.0062150.s006.docx]

**Table S**

|  | **Primer Sequence Forward** | **Primer Sequence Reverse** |
| --- | --- | --- |
| Olig2 | 5'-ATT ACA GAC CGA GCC AAC AC-3' | 5'-ACG ATG GGC GAC TAG ACA C-3' |
| GFAP | 5'-CCG CAT CAC CAT TCC TGT A-3' | 5'-GCA CAC CTC ACA TCA CCA C-3' |
| Sox2 | 5'-GGT TAC CTC TCC CTC CCA CTC CAG-3' | 5'-TCA CAT GTG CGA CAG GGG CAG-3' |
| Tuj1 | 5'-CAC CTA TTC AGG CCC GAC AA-3' | 5-CCT CCG TAT AGT GCC CTT TGG-3' |
| VEcad 3 | 5'-GGA CAG AGA AAC CTA CGC C-3' | 5'-CCT GCA CAA TGG ACT CTT TC-3' |
| End1 | 5'-GCA CCG GAG CTG AGA ATG G-3' | 5'-GTG GCA GAA GTA GAC ACA CTC-3' |
| End3 | 5'-CCC TGG TGA GAG GAT TGT GTA-3' | 5'-CCT TGT CCT TGT AAG TGA AGC AC-3' |
| VEGF-C | 5'-GTG AGG TGT GTA TAG ATG TGG GG-3' | 5'-ACG TCT TGC TGA GGT AAC CTG-3' |
| Nestin | 5'-CCC TGA AGT CGA GGA GTCG-3' | 5'-CTG CTG CAC CTC TAA GCG A-3' |
